# Monitoring the Cost and Affordability of a Healthy Diet within Countries: Building Systems in Ethiopia, Ghana, Malawi, Nigeria, Pakistan, Tanzania, and Viet Nam

**DOI:** 10.1016/j.cdnut.2024.104441

**Published:** 2024-08-20

**Authors:** Anna W Herforth, Rachel Gilbert, Kristina Sokourenko, Tehreem Fatima, Olutayo Adeyemi, Dawit Alemayehu, Eunice Arhin, Fantu Bachewe, Yan Bai, Imran Chiosa, Tirsit Genye, Hagos Haile, Raja Jahangeer, Joyce Kinabo, Fulgence Mishili, Chioma D Nnabugwu, John Nortey, Bernice Ofosu-Baadu, Adeyinka Onabolu, Daniel Bruce Sarpong, Masresha Tessema, Duong TT Van, Aishwarya Venkat, William A Masters

**Affiliations:** 1 Food Prices for Nutrition project, Friedman School of Nutrition Science and Policy, Tufts University, Boston, MA, United States; 2 FAO Pakistan, Islamabad, Pakistan; 3 World Bank, Islamabad, Pakistan; 4 Department of Human Nutrition and Dietetics, University of Ibadan, Ibadan, Nigeria; 5 Ethiopian Public Health Institute, Addis Ababa, Ethiopia; 6 Ministry of Food and Agriculture - Statistics, Research, and Information Directorate, Accra, Ghana; 7 International Food Policy Research Institute, Addis Ababa, Ethiopia; 8 Development Data Group, the World Bank, Washington, DC, United States; 9 Malawi National Statistical Office, Zomba, Malawi; 10 Ethiopian Statistical Service, Addis Ababa, Ethiopia; 11 Department of Human Nutrition and Consumer Studies, Sokoine University of Agriculture, Morogoro, Tanzania; 12 Global Alliance for Improved Nutrition, Abuja, Nigeria; 13 Ghana Statistical Service, Accra, Ghana; 14 Federal Ministry of Agriculture and Food Security, Abuja, Nigeria; 15 Department of Agricultural Economics and Agribusiness, University of Ghana, Legon, Accra, Ghana; 16 University of Medicine and Pharmacy, Ho Chi Minh City, Viet Nam; 17 Department of Agricultural Economics and Agribusiness, Sokoine University of Agriculture, Morogoro, Tanzania

**Keywords:** food security, food access, food prices, food price index, food-based dietary guidelines, food systems, Sustainable Development Goals

## Abstract

**Background:**

Governments around the world collect food price data on a frequent basis, often monthly, for the purpose of monitoring inflation. These routine economic data can be used with a nutrition-sensitive lens for understanding economic access to a healthy diet. The Food and Agriculture Organization of the United Nations has adopted the cost and affordability of a healthy diet (CoAHD) for annual tracking alongside other food security indicators. This indicator is relevant in many countries for informed decision-making and accountability toward Food Systems Summit pathways. National governments may wish to include this indicator in their own monitoring systems, using existing subnational price and income data.

**Objectives:**

We describe emerging systems in several countries for monitoring CoAHD and analytical tools that facilitate the calculation of CoAHD. We discuss reasons why the indicator may differ when calculated using subnational data compared with the global monitoring system and how to interpret differences.

**Methods:**

Between June 2016 and February 2024, 19 workshops were held in 7 countries (Ethiopia, Ghana, Malawi, Nigeria, Pakistan, Tanzania, and Viet Nam), where stakeholder discussions covered sources of food price data, institutions involved, policy uses, and direct training in calculation of CoAHD. Food price data collected by national organizations were used to calculate CoAHD in partnership with government agencies.

**Results:**

Calculating CoAHD using subnational data uses the same methods across settings, but the mechanisms for monitoring and dissemination are different in each country, illustrating heterogeneity in how the metric can most effectively be incorporated within existing structures. Results from national and global monitoring systems have expected differences based on data sources, healthy diet standards, and affordability standards.

**Conclusions:**

CoAHD can be calculated with existing data and resources, facilitated by new software tools and user tutorials. In the future, it can be further streamlined, leveraging technical assistance from global institutions and aligning national and global monitoring systems.

## Introduction

Governments around the world collect food price data on a frequent basis, often monthly or even more frequently, for the purpose of monitoring inflation. These routine economic data can be used with a nutrition-sensitive lens for understanding the cost of a healthy diet (CoHD) at retail prices. The FAO of the United Nations has adopted the cost and affordability of a healthy diet (CoAHD) as a global indicator of economic access to healthy diets, tracked alongside other indicators of food security. It is reported annually in the *State of Food Security and Nutrition in the World* report published by the United Nations food agencies and in databases curated by FAO and the World Bank [1,2].

CoAHD and associated indicators, including the cost of each food group recommended for daily consumption, are relevant in many countries for informed decision-making and accountability toward food systems summit commitments. National food systems transformation pathways documents in 95 countries include the theme of healthy diets from sustainable food systems [3]. CoAHD is an indicator of economic access to sufficient food to meet dietary needs for an active and healthy life. By highlighting the cost of total diets and the cost of each food group, it can guide agricultural production and food markets for improved nutrition. Monitoring CoAHD is crucial to food systems transformation agendas because it is the most direct outcome of changes in the food system, answering: have changes in the food system made it easier or harder for people to access healthy diets? As the global monitoring system reports only 1 national average (mean) figure using lagged food price data from a global dataset [4], national governments may wish to track CoAHD using their own official data that are more updated and granular than that which has been used in the global monitoring effort.

This paper describes the learnings from 8 y of engagement with government agencies toward calculating CoAHD. It provides an overview of the indicators and the software tools for facilitating their calculation using current food price data. We summarize the diverse learnings from engagement in each of the 7 countries in terms of data sources, dissemination platforms, policy applications, and interagency partnerships for monitoring CoAHD. Finally, we illustrate the differences between CoAHD using national and global processes using results from Pakistan as an example and discuss the reasons for the differences and interpretation of the results. In describing the process and results of working in countries to mainstream CoAHD monitoring by national governments, we distill conclusions and lessons learned to aid other countries where there is a desire to use CoAHD.

### Indicators and data inputs

The CoAHD refers to the minimum cost of purchasing a healthy diet in the market, defined as a diet that adheres to food-based dietary guidelines (FBDG), and the number and percent of people who cannot afford it. The FBDG are designed to promote healthy eating habits and prevent diet-related chronic diseases. A diet that adheres to FBDG is likely to meet nutritional needs, providing adequacy of essential macro- and micronutrients and a diversity of foods that reduce the risk of diet-related noncommunicable diseases while limiting salt, sugar, and other components of diet correlated with increased risk of noncommunicable diseases. Most FBDG identify a set of food groups, and many quantify amounts recommended for daily consumption.

The dietary standard used for CoAHD may differ between national and global CoHD monitoring systems. At the global level, the Healthy Diet Basket (HDB) [5,6] is used and developed to enable global comparisons based on average recommendations across many countries’ FBDG. It can readily be used as a healthy diet standard when countries do not have quantified FBDG. Within countries, national FBDG may be preferred as the dietary standard for CoAHD – if they have been developed and quantified – because they constitute a policy standard for how a healthy diet is defined for citizens.

Calculating the CoHD requires identifying the least-cost items in each food group recommended for daily consumption. The least-cost items are selected based on the cost of the amount required to satisfy daily food group recommendations. As consumers in markets readily perceive when selecting purchases, the specific items that are least expensive in each time and place will vary; for example, in August, mangos may be the least-cost fruit in a particular place, whereas in December, it may be oranges in the same place.

Food price data differ between national and global systems. For global monitoring, the calculation of CoHD uses the only dataset with prices across countries, compiled every 2–5 y by the international comparison program (ICP) at the World Bank [4]. The underlying price data are provided by national statistical offices (NSOs), but the ICP dataset has several limitations: having only 1 national average (mean) price per country for the year of observation, not necessarily capturing all of the commonly consumed food items for each country, forgoing items unique to specific countries because the purpose of the program is comparison across countries (which requires comparison of the same products); and being available only once every few years (most recently in 2017). Within countries, routinely collected price data are available that can show seasonal and subnational differences. In most countries, the NSO monitors retail prices for a range of locally available food items, typically 50–150 commonly consumed items, for tracking inflation with the consumer price index (CPI). These item prices are the official price data of the country, making these data the first choice for CoHD calculation within countries. The CPI items are sufficiently diverse to cover all food groups recommended in healthy diet standards. Other retail price data can also be used if they have sufficient diversity of items, such as retail prices from market information systems within ministries of agriculture or trade, which may have advantages in coverage of rural areas or more granular administrative levels.

A variant of the CoHD that accounts for food preferences (CoHD-FP) can be calculated by selecting more culturally preferred food items rather than least-cost food items. Cultural preferences are at the population level and do not refer to individual tastes. Population-level preferences are identified using household consumption or expenditure data in the population of interest to find the most commonly consumed foods within food groups. For example, there may be a preference for rice rather than millet, even if millet is cheaper, reflected in higher consumption and expenditure on rice.

In addition to the CoHD, the costs of each food group are useful indicators on their own. Food group costs can show the proportion of the CoHD commanded by each food group, as well as volatility or seasonal patterns in food group costs that might suggest specific needs for actions in agriculture or social protection for people to be able to afford a healthy diet. Costs of each food group can be tracked in absolute terms of least-cost items or as a unit-free index of all items in the food group; the latter is aligned with the food preferences variant of CoHD.

The affordability of a healthy diet is calculated by comparing the CoHD to an income standard. The cost of nonfood basic needs can be derived from the nonfood portion of poverty lines, or the amount that people near the poverty line spend on nonfood expenses (sourced from household survey data). After accounting for these basic nonfood expenses, income available for food is then compared with the CoHD. The percentage and number of people who cannot afford CoHD are reported by country, state, region, or other administrative unit.

## Methods

### Calculation of indicators

CoHD was developed and constructed to prioritize feasibility in low-income settings, so the computational complexity, data and software needs for calculating CoHD and cost of each food group are intentionally low. Calculating CoHD requires the following 3 types of data: *1*) retail food prices (cost per standard unit), *2*) food composition data on energy content and edible portion of food items, and *3*) quantified dietary guidelines (per person per day). Retail food price data can be obtained from a variety of sources, requiring only that the data are retail (not wholesale or farmgate) and contain a variety of foods in each food group. Data on energy content and edible portions of food items are available in various food composition tables [[7], [8], [9], [10]] and have been compiled for >400 items in the Food Item Information Database produced by the Food Prices for Nutrition Project [11].

Before starting the CoHD calculation, the healthy diet standard to be used must be quantified. National FBDG, if available, can be assessed to determine whether they are quantified (i.e, specifying amounts of each food group for daily consumption in terms of grams, proportion, or calories). If FBDG is unavailable or unquantified, the HDB can be used (Supplemental Table 1), which represents commonalities across FBDG and is used for global monitoring of CoHD [5,6]. When a national FBDG quantifies daily amounts of each food group in terms of weight or portion size, it is necessary to convert these quantities into kilocalories through the use of reference items (Supplemental Table 1). The total calories per day are then adjusted to match the global reference for the mean per capita energy requirement of 2330 kcal/d by scaling the quantities of each food group up or down proportionally. This step enables comparison across countries and with the global CoAHD dataset [[1], [2]].

The CoHD calculation process is as follows:

1) Classify each item in the food price dataset into a food group specified in the selected dietary standard: either national FBDG or the HDB (where the 6 food groups are starchy staples, vegetables, fruits, legumes, nuts or seeds, animal source foods, and oils and fats).
2) Match each food item in the food price dataset with a corresponding item in food composition tables to identify its energy density (kilocalories/gram) and edible portion. Item names may not match exactly; the key principle is to match the item that most closely corresponds in energy density and edible portion. It is not necessary to use food composition data specific to a country. The energy density and edible portion of food items do not vary systematically by nationality; variations in food composition from varieties, growing conditions, and storage affect the content of micronutrients and secondary compounds more than energy, and in any case, these can be as variable within countries as between them.
3) Calculate the cost per kilocalorie of each item (as sold) by dividing the item’s price (per kilogram) by its edible portion and then by its energy density (kilocalories/kilogram).
4) Identify the least-cost items in each food group in terms of cost per kilocalories. The number of items per food group is shown in Table 1 for the HDB, and similar numbers of foods can be selected per food group if using national FBDG for a total of 11–12 items.

**TABLE 1:** Summary of cost and affordability of a healthy diet inputs in each country seeking to monitor cost and affordability of a healthy diet.

Country	Agencies involved and steps taken toward systematizing CoAHD	Data source for retail food prices	Healthy diet standard	Interest and expected use	Dissemination platform(s)
Ghana	Co-creation of approach in 2016. Nationwide expansion of the food list monitored by the Ministry of Food and Agriculture (MoFA) since 2017. Monitoring efforts led by MoFA, in collaboration with Ghana Statistical Service (GSS), implementing Ghana’s first FBDG launched in 2023.	MoFA (prices), GSS (income)	Ghana FBDG (2023)	Implementation of new FBDG, food security tracking in MoFA	Planned: MoFA quarterly newsletter
Ethiopia	Collaborative effort between Ethiopian Public Health Institute (EPHI) and Ethiopian Statistical Service (ESS), using ESS data and EPHI’s FBDG and dissemination mechanism. Initial report published, newsletter in progress.	ESS	Ethiopia FBDG (2022)	Implementation of new FBDG, UN FSS goals	Planned: EPHI quarterly newsletter; Ethiopia FSD and NiPN
Malawi	Monitoring efforts led by the National Statistical Organization (NSO), with an initial report in progress.	Malawi NSO	HDB	Malawi Multi-Sectoral Nutrition Strategy	None yet
Nigeria	Collaborative effort with the National Bureau of Statistics (NBS) and GAIN country office, providing technical assistance to produce a monthly bulletin by request of NBS and incorporating CoAHD into Nigeria’s Food Systems Dashboard (FSD) and Governors’ Forum Nutrition Scorecard.	Nigeria NBS	HDB	Monitoring of food systems transformation and UN FSS goals, guidance on priority food groups and crops for agricultural policy support, early warning of populations vulnerable to food and nutrition insecurity, social protection, and minimum wage advocacy	NBS monthly report, Nigeria FSD, Nigeria Governors’ Forum Nutrition Scorecard
Pakistan	Collaborative effort with the Pakistan Bureau of Statistics (PBS) and FAO country office to produce a national POFI report with CoAHD. Future potential to incorporate CoAHD into Pakistan’s Food Systems Dashboard (FSD).	PBS	Pakistan FBDG (2018)	Pakistan POFI report, Pakistan FSD, Pakistan Food Security, and Nutrition Information System	Planned: PBS website; Pakistan POFI report; Pakistan FSD
Tanzania	Two workshops were held in 2016–2017 in the initial scoping study, with potential identified for monitoring by NBS.	Tanzania NBS	HDB	Relevant to 2021 UN FSS goals	None yet
Viet Nam	Results were produced with the collaboration of the General Statistics Office (GSO) and presented to GSO & National Institute of Nutrition (NIN) by an IMMANA postdoctoral associate.	Viet Nam GSO	Viet Nam FBDG (2016–2020)	Development of new FBDG, implementing NIN dietary guidance	None yet

Abbreviations: IMMANA, Innovative Methods, and Metrics in Agriculture and Nutrition Actions; NiPN, National Information Platform for Nutrition; POFI, Pakistan Overview of Food Security and Nutrition (FAO); UN FSS, United Nations Food Systems Summit.
5) Calculate the CoHD: Multiply the cost per calorie of each of the 11–12 least-cost items by the total calories needed of each food group (per person per day) divided by the number of items in each food group and sum the costs of all selected items to determine the total CoHD per person per day [6] (Supplemental Material).

A toolkit for the calculation of CoHD provides formulas and code that automate the calculation of CoHD [11].

CoHD-FP is calculated using the full food price list rather than selecting the least-cost items [[12], [13], [14]]. Food group shares are held constant in terms of kilocalories/day (as in Table 1 for HDB). Within each food group, the amount of each item in the dataset (in kcal) is in proportion to its share of consumption (or expenditure) within the total consumption (or expenditure) for the entire food group. An example using pseudodata is shown in Supplemental Table 2.

A simplified method of accounting for cultural food preferences in CoHD is to use the least-cost items among the most commonly consumed items in each food group; these are identified as the (*n* + 2) items in each food group with the highest consumption shares (if using household survey data) or expenditure shares (if using CPI data), where *n* = the number of items required in the HDB. Restricting the food list to this short list of culturally preferred food items, the CoHD can be calculated following the least-cost methods. An example using pseudodata is shown in Supplemental Table 2.

Additionally, a unit-free price index can be constructed for each food group recommended for daily consumption in healthy diets using standard CPI data and methods. Typically, each item in the CPI food list is coded with a Classification of Individual Consumption According to Purpose (COICOP) number [15]; these numbers can be used to automatically categorize each item into its HDB food group. In a few cases, the COICOP numbers are extended from a 6-digit to a 7-digit code needed for FBDG food group classification (Supplemental Table 2). Once items are classified by food group specified by HDB or FBDG, a price index is simply constructed for all the items in each food group using the same methods typically used for price indices [16]. These food group price indices can be used to monitor CoHD, given a baseline cost of each food group.

For CoAHD, calculating the number and percent of people who cannot afford a healthy diet requires 3 additional pieces of data: income data, for example, from the most recent household and expenditure survey data; an estimate of the cost of nonfood basic needs or expenditure on nonfood items; and the total population. Household total expenditure is often calculated by the institutions implementing the household survey and shared as a “consumption aggregate.” It is used as a measure of income in many countries, such as in contexts where cash incomes vary throughout the year and may be difficult for respondents to recall or where a substantial portion of the economy operates informally.

CoHD is unaffordable for people whose income is insufficient to purchase a least-cost healthy diet after nonfood basic needs are met. If the nonfood portion of the national or subnational poverty line is available, this amount (adjusted to the same period as the CoHD and income estimates) can be subtracted from total income per capita or adult equivalent. If the remaining income available for food is below CoHD, then CoHD is unaffordable. If the poverty line is not available or is not separated by food and nonfood needs, then household expenditure data can be used to find the median expenditure on nonfood (per capita per day) among households in the second income quintile in low- and lower-middle income countries (LMIC), as a proxy for the amount required for nonfood basic needs. This amount is subtracted from total income per capita. In LMIC, the nonfood spending among the second income quintile is expected to align more closely with meeting basic needs without including other nonessential expenditures that wealthier households might purchase. In the lowest income quintile, spending may be below the levels required to meet basic needs and, therefore, would not be an appropriate standard in LMIC. Given that expenditures and expenditure shares vary subnationally, countries can compute income available for food at lower geographic levels and compare that to CoHD in each geographic subunit. Affordability of a healthy diet is reported as the percentage of people (or number of people) who cannot afford CoHD at a given time and place.

### In-country workshops

Stakeholder meetings, trainings, and workshops were held in 7 African and Asian countries from 2016–2023: Ethiopia, Ghana, Malawi, Nigeria, Pakistan, Tanzania, and Viet Nam. The purpose of these meetings was to connect with local focal points in government agencies to understand relevant food price data, existing systems, capacity, and constraints and to discuss methods and reporting options among potential users. These meetings evolved from initial scoping studies in 2016–2017 and virtual stakeholder convenings in 2020–2021 to more involved cobuilding of CoAHD monitoring systems with government teams and national datasets in 2022–2024. Technical support provided by the Food Prices for Nutrition Project was adapted to each country’s circumstances, including available data, and tailored to the technical resources and software preferences of lead agencies. A list of workshops in each country is available in Supplemental Table 3. They were funded by the Bill and Melinda Gates Foundation and UKAid, with in-kind contributions and financial support from hosting and partner institutions in each country.

### Case study: calculation of CoAHD in Pakistan for within-country monitoring compared to global monitoring

In Pakistan, monthly price data from the Pakistan Bureau of Statistics (PBS) was used to calculate the cost of meeting Pakistan’s national FBDG, the Pakistan Dietary Guidelines for Better Nutrition, published by the Nutrition division of the Ministry of Planning, Development and Special Initiatives, Government of Pakistan [17]. This analysis used the complete list of monthly food price data collected by PBS, which includes 90 items for urban areas and 79 items for rural areas. The data were provided for each month from July 2019 to November 2023 as a district mean price for 35 urban and 27 rural districts.

The analysis was done using the CoHD software tools [11]. The Pakistan FBDG was quantified in partnership with the FBDG developers, and the amounts recommended per day were populated in the Excel workbook. Each priced item was matched to its respective food group in the Pakistan FBDG, and in the HDB for comparative analysis, as well as to food composition data to identify energy content and edible portion. CoHD was calculated twice, using the Pakistan FBDG and the HDB. The analysis was done in Stata.

Affordability data comes from the consumption and expenditure modules of the nationally and provincially representative 2018–2019 Household Integrated Economic Survey, also published by PBS. Data on food and nonfood expenditures were collected from 24,809 households, along with data on household composition, between August 2018 and June 2019. Expenditure data was adjusted for inflation to 2021 using urban and rural CPIs and compared with nominal CoHD in each month. Affordability was calculated first accounting for expenditures on nonfood identified from the Pakistan Household Integrated Economic Survey, aligned with the method used in global monitoring in 2024 [18]; and second, allocating 52% of income for food as was done in global monitoring in 2023 [19].

## Results

In 6 of the 7 countries, the primary data are sourced from the NSO from CPI data collection. Four of the 7 countries used national FBDG as their healthy diet standard (Ethiopia, Ghana, Pakistan, and Viet Nam) [17,[20], [21], [22]]; the remaining countries do not yet have quantified national guidelines and therefore used the HDB as a healthy diet standard. Data sources, healthy diet standards, dissemination platforms, involved institutions, and expected relevance/use of the indicators in each country are summarized in Table 1. The following section summarizes the learnings from each country.

### Ethiopia

A hybrid workshop was initially organized in early 2022, directly following the launch of Ethiopia’s first national FBDG. The Food Prices for Nutrition team, including the International Food Policy Research Institute (IFPRI) office in Addis Ababa, provided technical support to analysts from the Ethiopian Public Health Institute (EPHI) and the Ethiopian Statistical Service (ESS) for the calculation of CoHD, using Ethiopia’s FBDG as the healthy diet standard. The International Congress of Nutrition in December 2022 offered an opportunity for several stakeholders to reconvene in person and discuss data-sharing protocols among the institutions, process oversight, and integration of CoAHD indicators into existing national monitoring systems. EPHI, in collaboration with ESS, committed to leading national CoAHD analysis in January 2023 and co-organized an in-person training in Addis Ababa in April 2023 to develop a Stata-based process, address calculation challenges, and begin to develop a CoAHD bulletin for regular reporting. Later, in 2023, Ethiopia actively incorporated expenditure and wage data into the calculation process and became the first country government to publish results on CoAHD [23].

### Ghana

In Ghana, efforts to monitor CoAHD have involved stakeholders from the Ministry of Food and Agriculture (MoFA), the Ghana Statistical Service (GSS), and the University of Ghana. In 2016, Ghana was the first country where a national institution (MoFA) committed to using its data to calculate CoHD. After an initial scoping study on national data sources led by the first phase of the Food Prices for Nutrition Project (Indicators of Affordability of Nutritious Diets in Africa, IANDA), MoFA piloted an updated and expanded list of food items for its routine price data collection, after finding that it was missing sufficient diversity of foods to calculate CoHD, including several widely-consumed vegetable and animal source items. Finding that it added significant value without adding significant costs, MoFA institutionalized the updated food price monitoring list nationwide in 2017. An additional wave of meetings in 2022 reinvigorated discussions on the institutional roles and responsibilities for more routine CoAHD monitoring. In March 2023, an in-person workshop in Accra, co-organized with MoFA, piloted the calculation of the CoHD in Excel. Simultaneously, discussions with the GSS clarified the household survey data inputs for monitoring affordability. These parallel efforts coincided with the launch of Ghana’s first-ever national FBDG, which is used as the healthy diet standard for monitoring CoHD in Ghana. MoFA intends to publish CoHD in quarterly bulletins.

### Malawi

In Malawi, the Food Prices for Nutrition Project is collaborating with analysts from the NSO of Malawi. Preliminary CoAHD results were disseminated during an in-person multi-stakeholder workshop in Lilongwe in July 2023, where the NSO declared their ability to lead national efforts for CoAHD monitoring if it were of interest to stakeholders. Stakeholders from the Ministry of Health, research institutes, and development partners shared interest in using the findings to steer interventions. Preliminary training for calculating the CoHD in Excel was held at the NSO headquarters.

### Nigeria

In Nigeria, monitoring CoAHD has been advanced with support from the Global Alliance for Nutrition (GAIN) working closely with the National Bureau of Statistics (NBS). Awareness and interest in the indicator were sparked when stakeholders from academia, GAIN, and the Federal Ministry of Agriculture & Food Security attended a Food Prices for Nutrition learning laboratory at the Agriculture, Nutrition and Health Academy Week in 2018 in Accra, Ghana. Since that time, the Food Prices for Nutrition Project has provided technical support for the calculation of CoAHD. In 2021, Nigerian stakeholders identified the Nigeria subnational Food Systems Dashboard (FSD) as a dissemination platform for CoAHD. Researchers from Wageningen University and Research calculated CoAHD by state from the publicly available 2019 Nigeria Living Standards Survey [24]. In 2022, the Federal Ministry of Agriculture & Food Security and GAIN co-organized a preliminary stakeholder meeting on monitoring nutrition-sensitive food systems transformation in Nigeria for the presentation of results using survey data to NBS. A high-level meeting with NBS was organized in January 2023, where the NBS Statistician General appointed NBS focal points for the work using routine data, followed by a reconvening in-person in the International Conference on Agricultural Statistics held at the World Bank headquarters in May 2023, where the NBS Statistician General participated in a symposium on CoAHD. A hybrid learning event was held in June 2023 to share preliminary CoAHD results from routine data provided by NBS and learn more about NBS data management preferences. A September 2023 in-person workshop was subsequently held with NBS analysts in Abuja to cement inputs for regular CoAHD monitoring using Excel and explore options to disseminate findings through a monthly bulletin. Following the workshop, the Statistician General approved the monthly publishing of a CoHD Bulletin summarizing the preceding month’s CoHD in each state in Nigeria, with publications commencing January 2024. As Nigeria does not currently have a quantified national FBDG, the CoHD is calculated using the HDB. The cost in December 2023 was 786 Naira [25], which converts to $4.59 in 2022 purchasing power parity (PPP) [26]. This cost, using the same healthy diet standard but different price data, is in a similar range as the 2022 result of $3.83 reported in the *State of Food Security and Nutrition in the World 2024* report [18] and is plausible based on high inflation in 2023; however, the amounts are not directly comparable because the conversion factor from local currency with PPP has not yet been updated beyond 2022. CoAHD statistics calculated from the 2019 Nigeria Living Standards Survey are on the Nigerian FSD launched September 2023 and will be replaced in 2024 with indicators calculated from 2016 to 2023 NBS routine data. There are also plans in place to integrate CoAHD into the Nigeria Governors’ Forum Nutrition Scorecard. Media reports indicate that the NBS CoHD statistics were influential in negotiations with the federal government to set a new minimum wage that was ratified in July 2024.

### Tanzania

An initial scoping workshop was held in 2016, brought together stakeholders from multiple food price data collection agencies in the country and region; it was followed by another workshop in 2017, where the IANDA team shadowed food price data collection in major markets in Dar es Salaam. These meetings led to the determination that although multiple food price data exist, collected by different agencies and systems and covering different products and markets, the data most appropriate for monitoring CoAHD is from the NBS. Initial results using Tanzania NBS price data, showing the geographic and seasonal variation of CoAHD, were presented in the background paper to the *State of Food Security and Nutrition in the World 2020* report [12,27].

### Viet Nam

CoAHD has been calculated using Viet Nam’s FBDG as a healthy diet standard and food price data from the Viet Nam General Statistics Office (GSO) [28]. Results were presented to the GSO and National Institute of Nutrition by the lead author affiliated with the University of Medicine and Pharmacy at Ho Chi Minh City. The results show the feasibility and utility of analyzing GSO data routinely, which may be adopted by GSO if there is demand for the indicator.

### Pakistan: case study of differences between CoAHD produced in national compared with global monitoring

In Pakistan, the calculation and monitoring of CoAHD has been spearheaded by the FAO Country Office and supported by the PBS. An initial hybrid workshop in 2022 convened stakeholders from FAO, PBS, and several other national and international organizations, with the aim of raising awareness about current CoAHD methods and sparking discussions about roles and responsibilities for regular monitoring of these indicators. A Technical Committee was established shortly after the workshop to advise on national CoAHD calculations, and PBS data were used to calculate CoAHD to include in FAO’s *Pakistan Overview of Food Security and Nutrition* report — a national adaptation of the *State of Food Security and Nutrition in the World* report [18]. An in-person workshop in February 2024 focused on the routine use of PBS food price data for monitoring CoAHD, where stakeholders proposed to use both the PBS website and the recently launched Pakistan FSD [29] as a dissemination platform.

**FIGURE 1:**
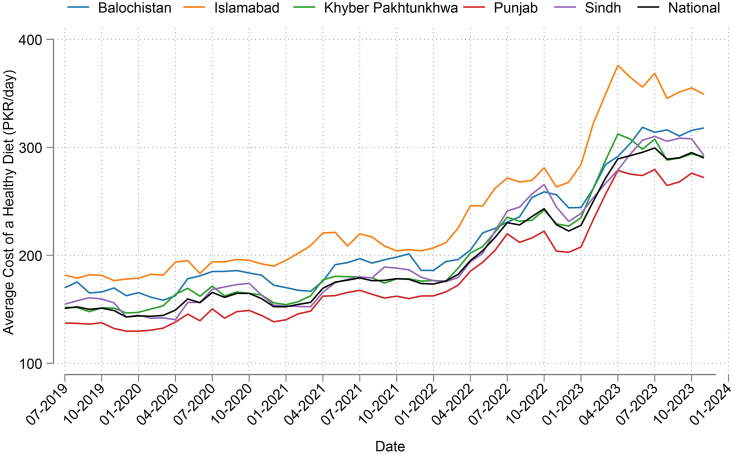
Spatial and temporal variation in the cost of a healthy diet in Pakistan, using Pakistan’s national food-based dietary guidelines [17]. PKR, Pakistani rupee.

Analysis of PBS monthly food price data allows monitoring of the CoHD, using Pakistan’s FBDG, by month and province (Figure 1). Table 2 provides a comparison among the different sources of food price data available for different purposes. Table 3 compares the CoHD estimated using the following 3 methods for comparison: *1*) the global monitoring method using ICP retail price data and the HDB [19]; *2*) PBS subnational retail price data and the HDB; *3*) PBS subnational retail price data and Pakistan’s national FBDG. The CoHD is lowest in case 2 [140 Pakistani rupees (PKR)] and highest in case 3, with a national mean cost of 171 PKR per person per day. Case 1, applying the global method, is 163 PKR, the cost published in the *State of Food Security and Nutrition in the World 2024* [18]. Quantification of Pakistan’s FBDG in comparison with the HDB, and a list of items most frequently identified as least-cost, are shown in Supplemental Tables 4 and 5 and Supplemental Figure 1.

**TABLE 2:** Comparison of food price data sources for global monitoring and within-country analyses.

Name	Coverage	Source	Advantages	Disadvantages
ICP	National average (mean) for a recent year	Data from national authorities assembled by ICP	Focus is global coverage (almost all countries and territories); high-quality control, fundamental for global economic data	Slow processing (data for 2021 became available in July 2024); only internationally comparable items
CPI	National and subnational averages (mean), often monthly	NSO data, typically published only in index form	Focus is a within-country pattern over all regions every month; high-quality control, official national price data	Item prices are often confidential; prices may not represent rural areas at lower administrative levels
MIS	Specific market locations, often monthly	Line ministries, often ministries of agriculture or trade	Focus is rural areas, designed to inform agricultural interventions	Data may not have sufficient diversity for CoHD calculation; may omit packaged foods such as oils
Ad-hoc	Specific items, vendors, and time periods	Programs and research	Focus is specific local zones or markets, which can be highly tailored to need	Prices obtained may not be comparable due to differences in data collection methods

Abbreviations: CoHD, cost of a healthy diet; CPI, consumer price index; ICP, International Comparison Program; MIS, market information system; NSO, national statistical office. Authors’ summary of data described in the text.

**TABLE 3:** Cost per day and cost share for each food group in 2021 Pakistani rupees, from analysis of the 2021 International Comparison Program (ICP) retail price data and Pakistan Bureau of Statistics (PBS) subnational retail food price data.

Food group	Result from global analysis using ICP data and HDB^1^		Pakistan’s national average (mean) using PBS data and HDB			Pakistan national average (mean) using PBS data and Pakistan FBDG	
	Cost share (%)	Cost per day (2021 PKR)	Cost share (%)	Cost per day (2021 PKR)		Cost share (%)	Cost per day (2021 PKR)
Starchy staples	12	20	16	22	Starchy staples	10	18
Oils and fats	6	12	7	10	Oils and fats	7	11
Fruits	27	34	17	24	Fruits	22	38
Vegetables	15	17	13	19	Vegetables	8	14
Legumes, nuts and seeds	10	14	10	14	Meat, pulses, and eggs	7	12
Animal source foods	30	48	37	51	Milk and milk products	46	78
**Total**	**100**	**145**	**100**	140	**Total**	**100**	**171**

1 Authors calculations based on cost of a healthy diet and food group costs for 2021, reported in FAOSTAT and World Bank DataBank [1,2]. These costs are reported in 2021 PPP and are converted to local currency units (PKR) using the 2021 PPP conversion factor for private consumption in Pakistan.

Table 4 shows the percentage of people who cannot afford a healthy diet using varied methods. Using PBS subnational price data, national FBDG as a healthy diet standard, and national affordability criteria in Pakistan leads to a total of 65% who cannot afford a healthy diet. This is slightly higher than the percentage using the HDB as a healthy diet standard (56%, Table 4), very similar to the figure published in the most recent global report (59%) [18]. Compared with estimates using global data sources and standards, in Pakistan, using subnational food price and income data slightly pushes down the estimate of the number of people who cannot afford it; conversely, the national FBDG (more expensive than HDB) pushes the number up. Previous methods for affordability using a fixed *proportion* of income for nonfood expenditures, rather than a fixed *amount*, estimated that more people (82%–83%) could not afford a healthy diet [19,30]. Expenditures on food and nonfood nationally and by province are shown in Supplemental Tables 6–8.

**TABLE 4:** Percent of people in Pakistan who cannot afford a healthy diet, 2021.

	2024 method with HDB^1^	2024 method with Pakistan FBDG^1^	2023 method with HDB^3^	2023 method with Pakistan FBDG^3^
	% who cannot afford a healthy diet Mean (SD)			
National	56	65	66	77
	(50)	(48)	(47)	(42)
Urban Khyber Pakhtunkhwa	50	63	62	78
	(50)	(48)	(48)	(42)
Rural Khyber Pakhtunkhwa	65	76	77	88
	(48)	(42)	(42)	(32)
Urban Punjab	33	41	41	55
	(47)	(49)	(49)	(50)
Rural Punjab^5^	58	66	68	79
	(49)	(47)	(47)	(41)
Urban Sindh	42	54	56	72
	(49)	(50)	(50)	(45)
Rural Sindh	81	87	90	95
	(39)	(33)	(29)	(21)
Urban Balochistan	68	75	78	88
	(47)	(43)	(41)	(32)
Rural Balochistan	86	91	93	97
	(35)	(29)	(25)	(17)

Abbreviations: CoHD, cost of a healthy diet; FBDG, food-based dietary guidelines; HDB, Healthy Diet Basket; PKR, Pakistani rupee; SD, standard deviation. Source: Authors calculations using the Pakistan 2018–2019 Household Integrated Economic Survey, adjusted using the consumer price index to real PKR in each month of 2021 for comparison with nominal CoHD in that same month.

1 The fixed amount reserved for nonfood expenses is determined as the median nonfood expenditure per adult equivalent among households in the second income quintile in terms of total expenditure. This is calculated at the provincial urban-rural level. A healthy diet is unaffordable if CoHD plus the fixed amount reserved for nonfood expenses is greater than total expenditures per adult equivalent per day. This method aligns with the method for calculating affordability used in the *State of Food Security and Nutrition in the World 2024* report.

3 This method defines a household as unable to afford a healthy diet if the CoHD is greater than their expenditure available for food (52% of the household’s total expenditure). Note, this affordability method was phased out after the *State of Food Security and Nutrition in the World 2023* report, in favor of using a fixed amount reserved for basic nonfood needs.

5 Islamabad is reported as part of Punjab in the 2018–2019 HIES data. No retail price data was specifically collected for rural Islamabad, so CoHD estimates for rural Punjab do not include rural Islamabad.

## Discussion

We discuss the differences between results in the Pakistan case study using subnational data and standards compared with published results from the global dataset and standards. We summarize lessons learned from countries’ steps toward implementing CoAHD nationally. Finally, we distill both of these into future directions for national and global monitoring of CoAHD.

### Comparison of results in global and national systems

As for many indicators, the national average used for cross-country monitoring is not exactly the same as the result using regularly updated subnational data. In the Pakistan case study presented here, the CoAHD was slightly higher when using subnational data sources and standards compared with the global dataset and standards. In Nigeria, the CoHD reported nationally in 2024 appears slightly higher than the CoHD reported in the SOFI in 2023 [19,25]. In other countries, the results may also differ, and not necessarily in the same direction. In summary, there are 3 primary reasons why CoAHD will differ between national and global monitoring systems:

#### Food price data

The use of subnational price data for calculating CoHD, such as CPI or market information system prices, is expected to generate lower estimates than what is found using the global ICP data. This expectation is based on the assumption that national price lists include more locally important, least-cost foods and more subnational variation in the data, which may include a wider variety of lower-cost markets, either urban or rural. The ICP food price data are collected for calculating PPP, so the food list reflects items that are regionally and globally important and comparable across countries. CPI food lists are based on items that are commonly consumed, usually determined by expenditure shares from household consumption and expenditure surveys.

#### Healthy diet standard

The costs of various FBDG are similar but not exactly the same; cost varies primarily based on differences in food groups recommended [5,12]. Animal source food groups are generally more expensive than plant-source, as is expected because they are at a higher trophic level and require more resources to produce. Therefore, FBDG that recommend more animal source foods (either in quantity and/or specific food groups that do not contain plant-source substitutes) are generally more expensive. We see this in the Pakistan FBDG compared with the HDB, as well as similar results in Viet Nam. Where the use of national FBDG revealed high-cost animal source food requirements, the policy implications of these results were a point of consideration, especially where dairy is not traditionally part of diet patterns (Viet Nam).

#### Income data and affordability standard

The share of expenditure on food varies considerably across countries and, in many cases, between different socioeconomic groups within countries. The Pakistan case study shows that the expenditures of the second income quintile are very close to those within 20% of the Pakistan poverty line – indicating that the expenditures of the second quintile are a proxy for what has been previously defined as the cost of basic needs. Their nonfood expenditures can be used as an amount reserved for nonfood expenses and added to the CoHD to determine affordability. Using country-specific mean expenditures among a representative group whose income is close to the poverty line will produce a different, more country-specific affordability standard than the global standard method. Furthermore, CoHD can be computed at the subnational level, where it is more expensive in some regions compared to others and compared with subnational income, as shown in the case study. Summing the number who cannot afford CoHD in each region may result in a different national total than finding the national mean CoHD and the number who cannot afford it.

### Lessons learned toward integrating CoAHD into national monitoring systems

In most countries, the NSO coordinates calculation, but the motivation to calculate the indicator lies in other ministries. One NSO director remarked that the NSO has the capability of producing CoAHD but will only do so based on demand from other stakeholders.

Interest across agriculture, labor, and health sectors has driven the demand for frequent, subnational monitoring of the CoAHD indicators. In Ghana, MoFA saw the direct value of the indicator toward better use of their own data for their mandate of monitoring food security and as well as agribusiness opportunities to fill gaps in food access. In Ethiopia, as a complement to the affordability statistic, unskilled daily labor wages are presented as a ratio to CoHD, which may have implications around the living wage.

Demand for the indicators has been strong in countries with new FBDG. In Ethiopia and Ghana, the countries’ first FBDGs were launched in 2022–2023, and the CoAHD served as part of their implementation. Traditionally, implementation of FBDG is considered to consist of nutrition education and information campaigns. This new generation of FBDG developers sees implementation as primarily a matter for ministries of food, agriculture, social protection and education – to make the diets recommended in the guidelines available and affordable to all. Thus, FBDG is a guide for food systems transformation, and the CoAHD, therefore, serves an important function in translating dietary guidance into action areas for market development, diversification of agriculture, and social protection.

Collaborative multistakeholder efforts involving other government agencies, research institutions, and international organizations (such as FAO, GAIN, and IFPRI) have played a pivotal role in the production and use of CoAHD as institutionalized indicators of food security. New efforts have also been made by the World Food Programme (WFP), with the collaboration and technical support of the Food Prices for Nutrition Project, to add CoAHD to their ongoing, similarly-named “Cost of the Diet” analyses so that future WFP analyses could include both least-cost nutrient-adequate and least-cost healthy diets [31].

The primary dissemination platform used by countries is a monthly or quarterly brief published by a government agency. In Nigeria and Pakistan, the publisher is the NSO [25], whereas in Ethiopia, it is the Public Health Institute (part of the Ministry of Health), and in Ghana, it is the MoFA. The need for online accessibility has been emphasized by all. In several countries, secondary dissemination platforms have provided further motivation to fill data gaps and effectively disseminate information integrated with other relevant data. These include the subnational FSD in Nigeria and Pakistan and the National Information Platform for Nutrition in Ethiopia. In these cases, the secondary platforms are being designed to pull data from the original government source to ensure accuracy and coordination. The inclusion of the CoAHD in these platforms helps to link it to other food systems and nutrition indicators (e.g., availability of different food groups, diet quality, and nutritional status) for use in diagnosing challenges and informing policy and programs [32,33].

A key outcome of 8 y of workshops is the global public good of streamlined methodological tools to calculate CoAHD. The development of the CoHD toolkit [11] was based on feedback and observations about equipment, software, and analytical capacity during stakeholder meetings and training workshops. It was developed for Microsoft Excel, Stata, and R software widely used by NSOs and research institutions, including all those reported here. The CoHD toolkit simplifies the process of calculating the CoHD using countries’ own retail prices, accommodating a small or large list of items and different levels of granularity of food price data in terms of frequency (monthly, yearly), and location (market, city, district, state or region).

To automate the calculation of CoHD, users enter prices into the workbook, convert them into prices per kilogram, select the dietary standard they wish to use (national FBDG or global HDB) and follow a set of instructions (written or via a video tutorial) to match the food and beverage items in their data to the correct food groups and food composition data in the workbook. For an NSO or other user with repeated price observations for the same food list, this matching process needs only to be performed once, streamlining the CoHD calculation process for each additional round of food price data (e.g., for NSOs seeking to calculate the indicator monthly with the same food list). The Excel workbook formulas automatically compute the CoHD once all inputs are complete. Users can also choose to use the Stata or R code, which uses the dietary standard and food composition matching sheets from the Excel workbook as data input.

For many users (who are not nutritionists), the largest challenge in the process was finding food composition data to identify the energy content and edible portion of each food item, which is necessary to select the cheapest items within each food group on a cost-per-calorie basis. Therefore, the “Food Item Information Database” was included in the toolkit, which compiles the needed food composition data for >400 items found in NSO food price datasets. Another step that was difficult for users was quantifying their national FBDG into daily amounts of each food group recommended. Therefore, the Excel workbook is prepopulated with target quantities (kilocalories/food group/day) for the global HDB and a diverse list of quantified national FBDG so users can select the most appropriate healthy diet standard for their calculation [34].

Affordability calculations are not automated due to the complexity and variability of household survey and inflation data. The original CoHD-FP variant is not automated, but the simplified food preferences variant can readily be computed using the toolkit if the only price data entered into the workbook are those items with the highest expenditure shares in each food group.

### Future directions

Although the calculation of CoHD is not technically challenging and requires no new data collection, as a new indicator, it requires new systems for data flows, time from personnel, regular reporting, and an impactful outlet for dissemination. No matter how simple the indicator, each of these requires time and commitment. During the Food Prices for Nutrition Project period, staff turnover in collaborating institutions was fortunately low, and the same focal points were able to advance the methods over a period of several years. This is not always the case, however, and new methods are vulnerable to staff turnover if too few people are trained in and committed to them.

In the future, global and national monitoring may be more closely aligned if updated national price data are made available for updated CoHD figures tracked by the global agencies (FAO, World Bank). Also, identifying and training staff in each country is labor intensive. A more efficient way may be to systematize price reporting and calculate centrally in global institutions using HDB, which would then be used by both national and global institutions. Country ministries or agencies may still wish to implement their own systems reflecting national FBDG or data from other ministries (e.g., agriculture), and the CoHD software toolkit will remain available for national and other users.

## Acknowledgments

We thank Anna Lartey, Richmond Aryeetey, and the Institute of Statistical Social and Economic Research group at the University of Ghana for their enthusiasm and support in convening conversations, as well as support in using and interpreting the new Ghana food-based dietary guidelines (FBDG). Daniel Mekonnen, Wageningen University and Research (WUR), for collaboration on the initial Nigeria analysis using household survey data. Elise Talsma, WUR, for motivating the work in Viet Nam. Shelly Sundberg, Bill & Melinda Gates Foundation, for advocating for grounding the work in countries toward transforming agriculture and food systems. Jennifer Coates, Zak Gersten, and Julia Matteson, Tufts University, for providing support and input into the workshops and the many other collaborators from the Indicators of Affordability of Nutritious Diets in Africa (IANDA) and Changing Access to Nutritious Diets in Africa and South Asia (CANDASA) projects. Joe Yates, Suneetha Kadiyala, and ANH Academy Week organizers for the opportunity to hold the CoAHD learning laboratories for 8 y (and counting), offering multiple opportunities to connect with interested stakeholders in many countries. GAIN staff for coordinating workshops in Nigeria and Pakistan with the Food Systems Dashboard, Ramani Wijesinha Bettoni, and Carlo Cafiero, FAO, for feedback on Pakistan FBDG and affordability analysis.

## Author contributions

The authors’ responsibilities were as follows – AWH, RG, KS: designed and conducted the research, analyzed data, and wrote the paper; YB, RJ, DBS, WAM: designed the research; TF, DA, OA, IC, DTTV, TG, AV: conducted analyses; TF: conducted analyses for the Pakistan case study; EA, HH, RJ, JK, FM, CDN, JN, BO-B, AO, DBS, MT: provided essential materials; AWH: had primary responsibility for final content; WAM, AWH: obtained funding; and all authors: read and approved the final manuscript.

## Conflict of interest

The authors report no conflicts of interest.

## Funding

This work was supported by a grant from the Bill & Melinda Gates Foundation and the Foreign, Commonwealth and Development Office of the United Kingdom government for Food Prices for Nutrition (2020–2024), INV-016158, by a grant from the Bill & Melinda Gates Foundation and UKAid for CANDASA (2017-2020), OPP1182628; and by an IMMANA grant funded by UKAid for IANDA (2015-2017). In-kind and/or financial support for in-country workshops was provided by the Ethiopian Public Health Institute (Ethiopia 2023, 2024); FAO Pakistan (Pakistan 2022, 2024); the GAIN (Pakistan 2024), in Nigeria supported by a grant from the Bill & Melinda Gates Foundation (Nigeria 2022, 2023); The World Bank (Malawi 2023). The supporting sources had no involvement in the study design, collection, analysis, and interpretation of data, writing of the report, nor restrictions regarding the submission of the report for publication.

## Data availability

Data on the cost and affordability of a healthy diet at the global level is publicly and freely available without restriction at https://www.fao.org/faostat/en/#data/CAHD and https://www.worldbank.org/foodpricesfornutrition. The underlying retail price data from the international comparison program is available upon request from the World Bank. Country price data are available upon request from national statistical offices or other data-collecting agencies in the relevant countries. All household survey data used is publicly available.

## Appendix A Supplementary data

Multimedia component1 Multimedia component1
