# Supplementary material for: Monitoring the Cost and Affordability of a Healthy Diet within Countries: Building Systems in Ethiopia, Ghana, Malawi, Nigeria, Pakistan, Tanzania, and Viet Nam
Source: Curr Dev Nutr. 2024 Aug 20;8(10):104441. doi: 10.1016/j.cdnut.2024.104441 (PMC11490755; doi:10.1016/j.cdnut.2024.104441)
Supplement: Multimedia component1 [file mmc1.docx]

# Supplementary materials

**Supplementary Table 1.** Healthy Diet Basket content by food group: Average food group amounts recommended across FBDG scaled to meet a consistent dietary energy intake target (2,330 kcal)

| **Food group** | **Number of food items selected for cost of healthy diet** | **Total energy content (kcal)** | **Equivalent gram content, by reference food (edible portion)** |
| --- | --- | --- | --- |
| Starchy staples | 2 | 1 160 | 322 g dry rice |
| Vegetables | 3 | 110 | 270–400 g vegetables |
| Fruits | 2 | 160 | 230–300 g fruits |
| Animal source foods | 2 | 300 | 210 g egg |
| Legumes, nuts & seeds | 1 | 300 | 85 g dry bean |
| Oils & fats | 1 | 300 | 34 g oil |

Note: Different food items have different calories per gram. To equate calories and grams, the following reference food items were used: dry rice for starchy staples; egg for animal source foods; dry bean for legumes, nuts and seeds. For fruits and vegetables, the range is based on the lowest to highest kcal/g across the International Comparison Program (ICP) dataset for each. For food-based dietary guidelines (FBDG) that recommend daily consumption of dairy as a separate group, the fresh whole milk can be used as the reference food. For FBDG that recommend daily consumption of nuts and seeds as a separate food group, peanuts can be used as the reference food item.

Source: Herforth et al. 2023.^1^

**Supplementary Material: Calculation of CoHD** (Source: Herforth et al. 2023)^[[1]](#footnote-1)^

${Cost}_{StarchyStaples}=min\{\sum_{i=1}^{2} p_{i}q_{i}\}, where each q_{i}=580 kcal \left( =\frac{1160}{2} \right)and item i is a starchy staple$ (1)

${Cost}_{AnimalFoods}=min\{\sum_{i=1}^{2} p_{i}q_{i}\}, where each q_{i}=150\mathrm{kcal} \left( =\frac{300}{2} \right)and item i is an animal source food$ (2)

${Cost}_{LegsNutsSeeds}=\min\left\{ p_{i}q_{i} \right\}, where each q_{i}=300 kcal and item i is a legume, nut or seed$ (3)

${Cost}_{Vegetables}=min\{\sum_{i=1}^{3} p_{i}q_{i}\}, where each q_{i}=66.7 \mathrm{kcal}\left( =\frac{110}{3} \right)and item i is a vegetable$ (4)

${Cost}_{Fruits}=min\{\sum_{i=1}^{2} p_{i}q_{i}\}, where each q_{i}=80 \mathrm{kcal}\left( =\frac{160}{2} \right)and item i is a fruit$ (5)

${Cost}_{OilsFats}=\min\left\{ p_{i}q_{i} \right\}, where each q_{i}=300 kcal and item i is a lipid$ (6)

The total cost of a healthy diet is calculated by summing costs across food groups, as follows:

${Cost}_{HealthyDiet}=\sum_{j=1}^{6} {Cost}_{j}, for all j=\left\{ 1, \ldots, 6 \right\} food groups$ (7)

**Supplementary Table 2.** Example of COICOP codes for items within group foods; Selection of items in a restricted food list of culturally preferred items for CoHD; and CoHD-FP (food preferences) composition, showing calculation for kcal of each item

| **COICOP-N^1^** | **COICOP** | **Item Name** | Food group | CPI weight | Selected for restricted food list of culturally preferred items^2^ | kcal in CoHD-FP (in this example list; see calculation) | Calculation for kcal in CoHD-FP (where wt_i = the CPI weight of item i, which is divided by the sum of the CPI weights of all items in the food group of item i) |
| --- | --- | --- | --- | --- | --- | --- | --- |
|  | 01.1.7.5.4 | Yam | Starchy staples | **4.19** | x | 219 | 1160*(wt_i/(SUM_SSwt) |
|  | 01.1.1.1.2 | Rice | Starchy staples | **4.16** | x | 218 | 1160*(wt_i/(SUM_SSwt) |
|  | 01.1.7.5.3 | Cassava | Starchy staples | **3.81** | x | 200 | 1160*(wt_i/(SUM_SSwt) |
|  | 01.1.1.1.5 | Millet | Starchy staples | **2.63** | x | 138 | 1160*(wt_i/(SUM_SSwt) |
|  | 01.1.1.1.9 | Oats | Starchy staples | 2.44 |  | 128 | 1160*(wt_i/(SUM_SSwt) |
|  | 01.1.1.1.6 | Maize | Starchy staples | 1.99 |  | 104 | 1160*(wt_i/(SUM_SSwt) |
|  | 01.1.1.2.1 | Wheat Flour | Starchy staples | 1.47 |  | 77 | 1160*(wt_i/(SUM_SSwt) |
|  | 01.1.7.5.7 | Plantain | Starchy staples | 1.44 |  | 76 | 1160*(wt_i/(SUM_SSwt) |
|  | 01.1.7.2.4 | Tomatoes | Vegetables | **5.21** | x | 27 | 110*(wt_i/(SUM_Vegwt) |
|  | 01.1.7.4.3 | Onions | Vegetables | **4.46** | x | 23 | 110*(wt_i/(SUM_Vegwt) |
|  | 01.1.7.2.3 | Eggplant | Vegetables | **3.68** | x | 19 | 110*(wt_i/(SUM_Vegwt) |
|  | 01.1.7.1.5 | Spinach | Vegetables | **2.90** | x | 15 | 110*(wt_i/(SUM_Vegwt) |
|  | 01.1.7.2.1 | Green pepper | Vegetables | **2.55** | x | 13 | 110*(wt_i/(SUM_Vegwt) |
|  | 01.1.7.1.9 | Amaranth leaves | Vegetables | 2.12 |  | 11 | 110*(wt_i/(SUM_Vegwt) |
|  | 01.1.6.1.7 | Pineapple | Fruit | **3.88** | x | 38 | 160*(wt_i/(SUM_fruitwt) |
|  | 01.1.6.1.8 | Coconut | Fruit | **3.73** | x | 37 | 160*(wt_i/(SUM_fruitwt) |
|  | 01.1.6.1.5 | Mango | Fruit | **3.51** | x | 34 | 160*(wt_i/(SUM_fruitwt) |
|  | 01.1.6.3.1 | Apples | Fruit | **2.56** | x | 25 | 160*(wt_i/(SUM_fruitwt) |
|  | 01.1.6.2.3 | Oranges | Fruit | 1.42 |  | 14 | 160*(wt_i/(SUM_fruitwt) |
|  | 01.1.6.1.2 | Banana | Fruit | 1.23 |  | 12 | 160*(wt_i/(SUM_fruitwt) |
|  | 01.1.6.8.8 | Groundnuts (shelled) | Legumes, nuts & seeds | **2.54** | x | 145 | 300*(wt_i/(SUM_LNSwt) |
|  | 01.1.7.6.6 | Cowpeas | Legumes, nuts & seeds | **1.65** | x | 94 | 300*(wt_i/(SUM_LNSwt) |
|  | 01.1.7.6.2 | Fava beans | Legumes, nuts & seeds | **1.07** | x | 61 | 300*(wt_i/(SUM_LNSwt) |
|  | 01.1.2.2.1 | Beef with bones | Animal-source foods | **2.31** | x | 64 | 300*(wt_i/(SUM_ASFwt) |
|  | 01.1.3.2.9 | Dried fish | Animal-source foods | **2.07** | x | 58 | 300*(wt_i/(SUM_ASFwt) |
|  | 01.1.4.8.1 | Eggs | Animal-source foods | **1.51** | x | 42 | 300*(wt_i/(SUM_ASFwt) |
|  | 01.1.4.3.2 | Powdered milk | Animal-source foods | **1.40** | x | 39 | 300*(wt_i/(SUM_ASFwt) |
|  | 01.1.2.2.4 | Chicken (fresh) | Animal-source foods | 1.31 |  | 36 | 300*(wt_i/(SUM_ASFwt) |
|  | 01.1.2.2.3 | Goat (fresh) | Animal-source foods | 1.23 |  | 34 | 300*(wt_i/(SUM_ASFwt) |
|  | 01.1.3.3.1 | Tuna in vegetable oil | Animal-source foods | 0.95 |  | 26 | 300*(wt_i/(SUM_ASFwt) |
|  | 01.1.5.1.2 | Palm oil | Oils and fats | **2.05** | x | 108 | 300*(wt_i/(SUM_OFwt) |
|  | 01.1.5.1 | Vegetable oil | Oils and fats | **1.83** | x | 96 | 300*(wt_i/(SUM_OFwt) |
|  | 01.1.5.1.5 | Groundnut oil | Oils and fats | **1.03** | x | 54 | 300*(wt_i/(SUM_OFwt) |
|  | 01.1.5.1.6 | Coconut oil | Oils and fats | 0.79 |  | 42 | 300*(wt_i/(SUM_OFwt) |
|  | 01.2.6.0.0 | Coca Cola /Fanta (bottle) | Discretionary | 1.08 |  |  |  |
| 01.1.1.3.9.b | 01.1.1.3.9 | Biscuit (simple cookie) | Discretionary | 0.77 |  |  |  |
|  | 01.1.8.1 | Granulated sugar | Discretionary | 0.73 |  |  |  |
|  | 01.1.8.6.0 | Ice cream | Discretionary | 0.38 |  |  |  |
|  | 01.1.9.1.6 | Instant noodles | Discretionary | 0.34 |  |  |  |
| 01.2.1.0.0.a | 01.2.1.0.0 | Fruit Juice | Discretionary | 0.20 |  |  |  |
| 01.1.7.2.1a | 01.1.7.2.1 | Chilies (pepper) | Excluded | 0.57 |  |  |  |
|  | 01.1.9.3.1 | Salt (iodised) | Excluded | 0.51 |  |  |  |
|  | 01.2.3.0.9 | Tea bags (eg. Lipton) | Excluded | 0.31 |  |  |  |
|  | 01.2.5.0.0 | Mineral water (bottled) | Excluded | 0.30 |  |  |  |
|  | 01.2.2.0.1 | Coffee (ground) | Excluded | 0.24 |  |  |  |
|  | 01.1.7.4.2 | Garlic | Excluded | 0.21 |  |  |  |
|  | 01.1.7.4.9 | Ginger | Excluded | 0.15 |  |  |  |
|  |  | TOTAL |  |  | 23 items, of which 11 will be identified as least-cost at any given time | All items in HDB food groups, 2330 kcal |  |

^1^ COICOP-N suffixes provide information for classifying items into food groups recommended for health, where COICOP codes are insufficient.

^2^ In each food group, (n+2) items with the highest consumption shares are retained in the restricted food list, where n = number of items required in the Healthy Diet Basket (HDB).

**Supplementary Table 3.** In-country workshops on Cost and Affordability of a Healthy Diet

| **Country** | **Dates and mode of workshops** |
| --- | --- |
| Ghana | In person (Apr 21, 2016)  In person (Aug 8, 2017)  In person (Jun 27, 2018)  Virtual (Sept 20, 2022)  In person (Mar 27-31, 2023)  One on one online meetings with lead analysts. |
| Ethiopia | Hybrid (Jun 7-9, 2022)  In person (Apr 3-7, 2023) In person (Feb 12-16, 2024)  One on one online meetings with lead analysts. |
| Malawi | In person (Jul 3-5, 2023)  One on one online meetings with lead analysts. |
| Nigeria | Hybrid (Aug 17-18, 2022)  Hybrid (Jan 17, 2023)  Hybrid (Jun 14-15, 2023)  In person (Sept 25-29, 2023)  Hybrid (Jan 31, 2024)  One on one online meetings with lead analysts. |
| Pakistan | Hybrid (Feb 15-16, 2022)  In person (Feb 19-23, 2024)  One on one online meetings with lead analysts. |
| Tanzania | In person (Jun 28, 2016)  In person (Aug 11, 2017) |
| Viet Nam | In person (Feb 6, 2023)  One on one online meetings with lead analyst. |

Materials (slides and/or meeting reports) from workshops available at: https://sites.tufts.edu/foodpricesfornutrition/countries/

**Supplementary Table 4.** Quantification of Pakistan FBDG and Healthy Diet Basket

| **Healthy diet standard** | **Food Group** | **Number of foods recommended** | **Calories  (kcal/day)** |
| --- | --- | --- | --- |
| Pakistan food-based dietary guidelines | Starchy staples | 2 | 927 |
|  | Oils and fats | 1 | 330 |
|  | Fruits | 2 | 258 |
|  | Vegetables | 3 | 80 |
|  | Meat, pulses, and eggs | 2 | 256 |
|  | Milk and milk products | 1 | 479 |
|  |  |  |  |
| Healthy Diet Basket | Starchy staples | 2 | 1160 |
|  | Oils and fats | 1 | 300 |
|  | Fruits | 2 | 160 |
|  | Vegetables | 3 | 110 |
|  | Legumes nuts and seeds | 1 | 300 |
|  | Animal source foods | 2 | 300 |

#

**Supplementary Figure 1.** Cost shares in quantified Pakistan food-based dietary guidelines (FBDG) and Healthy Diet Basket, 2021

A. Pakistan FBDG

B. Healthy Diet Basket

Note: National average of all food group cost shares from monthly urban-rural province costs, 2021.

| **Supplementary Table 5.** Least-cost foods commonly identified in Pakistan, from the 2017 International Comparison Program (ICP) prices and from retail food price data from Pakistan Bureau of Statistics (PBS) |
| --- |

| **Healthy Diet Basket food group** | **Least-cost items identified  (2017 ICP)** | **Commonly identified least-cost items (subnational retail price data from PBS, 2017-2021)** |
| --- | --- | --- |
| Starchy staples | Wheat flour | Wheat |
|  | Maize | Rice IRRI-6/9 (Sindh/Punjab) |
| Oils and fats | Vegetable oil | Mustard Oil |
| Fruits | Bananas | Bananas (Kela) Local |
|  | Coconut, young green | Guava (Amrood) |
| Vegetables | Onions | Onions |
|  | Carrots | Carrots (Gajar) |
|  | Water spinach | Turnip (Shalgham)* |
| Legumes nuts and seeds | Pulse Masoor | Pulse Masoor (Washed) |
| Animal source foods | Buffalo milk, unpasteurized | Milk fresh (Un-boiled) |
|  | Chicken (Live) | Chicken Farm Broiler (Live) |
| *Turnip is classified as a vegetable in Pakistan’s food-based dietary guidelines (FBDG), although it is classified as a starchy staple in the Healthy Diet Basket (HDB). | | |

| **Supplementary Table 6.** Average share of expenditure on food among different reference populations, Pakistan, 2021 | | | | |
| --- | --- | --- | --- | --- |
|  | **All HH** | **Q1 HH**^1^ | **Q2 HH**^1^ | **HH within 20% of poverty line**^2^ |
|  | % of total expenditure spent on food  Mean (SD) | | | |
| National | 44.1 | 50.8 | 47.8 | 48.2 |
|  | (11.8) | (10.0) | (9.8) | (9.9) |
| Urban Khyber Pakhtunkhwa | 41.5 | 50.4 | 46.7 | 46.9 |
|  | (11.6) | (9.0) | (8.9) | (9.1) |
| Rural Khyber Pakhtunkhwa | 44.9 | 49.7 | 46.9 | 47.3 |
|  | (11.2) | (9.3) | (8.9) | (8.9) |
| Urban Punjab | 37.8 | 45.7 | 44.4 | 44.1 |
|  | (11.2) | (10.4) | (9.6) | (10.0) |
| Rural Punjab | 44.8 | 48.4 | 46.9 | 47.3 |
|  | (10.9) | (9.3) | (9.8) | (9.7) |
| Urban Sindh | 41.8 | 51.8 | 47.5 | 48.0 |
|  | (10.7) | (9.1) | (9.5) | (9.4) |
| Rural Sindh | 52.1 | 55.9 | 51.8 | 52.5 |
|  | (10.3) | (9.2) | (9.2) | (9.5) |
| Urban Balochistan | 45.7 | 52.2 | 48.9 | 49.2 |
|  | (11.6) | (10.2) | (9.6) | (10.0) |
| Rural Balochistan | 51.2 | 54.8 | 51.7 | 52.1 |
|  | (11.4) | (10.1) | (9.9) | (10.2) |
| Source: Authors calculations using the 2018-19 Pakistan Household Integrated Economic Survey (HIES).  ^1^ Households in the first (Q1) or second (Q2) quintile in terms of total expenditure. Quintiles are computed using total expenditure at the national level and household sampling weights.  ^2^ Households whose expenditures are within 20 percent of the 2015-16 poverty line = 3250 PKR per adult equivalent per month. | | | | |

| **Supplementary Table 7.** Expenditure available for food using different methods, Pakistan, 2021 | | | | |
| --- | --- | --- | --- | --- |
| **Reference group for food expenditure share (or share used for calculating available food budget)** | **Total income minus fixed non-food expenses** | **Provincial urban/rural median shares of Q2 HH**^1^ | **Provincial urban/rural median shares of those within 20% of the poverty line**^2^ | **SOFI 2023 method (52% of total expenditure)**^3^ |
|  | **Expenditure available for food (PKR/day/adult equivalent)**  **Mean (SD)** | | | |
| National | 202 | 137 | 138 | 153 |
|  | (257) | (117) | (118) | (134) |
| Urban Khyber Pakhtunkhwa | 220 | 147 | 147 | 162 |
|  | (223) | (105) | (105) | (116) |
| Rural Khyber Pakhtunkhwa | 165 | 120 | 120 | 133 |
|  | (148) | (69) | (70) | (77) |
| Urban Punjab | 297 | 174 | 174 | 205 |
|  | (331) | (146) | (146) | (172) |
| Rural Punjab | 174 | 124 | 124 | 138 |
|  | (170) | (80) | (80) | (89) |
| Urban Sindh | 276 | 172 | 175 | 192 |
|  | (447) | (208) | (212) | (232) |
| Rural Sindh | 122 | 106 | 107 | 107 |
|  | (104) | (54) | (54) | (54) |
| Urban Balochistan | 167 | 124 | 124 | 133 |
|  | (154) | (75) | (75) | (80) |
| Rural Balochistan | 120 | 103 | 105 | 105 |
|  | (92) | (47) | (48) | (48) |
| Source: Authors calculations using the 2018-19 Pakistan Household Integrated Economic Survey (HIES). The poverty line information is found in the World Bank “Poverty and Equity Brief: Pakistan”, April 2020.  ^1^ Households in the second quintile in terms of total expenditure. Quintiles are computed using total expenditure at the national level and household sampling weights. Expenditure available for food is calculated as average share of expenditure on food for this reference group times households’ total expenditure per adult equivalent.  ^2^ Households whose expenditures are within 20 percent of the 2018-19 poverty line = 3776 PKR per adult equivalent per month. Expenditure available for food is calculated as average share of expenditure on food for this reference group times households’ total expenditure per adult equivalent.  ^3^ Expenditure available for food is calculated as 52% of household’s total expenditure per adult equivalent among all households. Note, this affordability method was phased out after the *State of Food Security and Nutrition in the World 2023* report (SOFI), in favor of using a fixed amount reserved for basic non-food needs. The updated approach is shown in the first column. | | | | |

| **Supplementary Table 8.** Average non-food expenditure for different sub-groups, Pakistan, 2021 | | | |
| --- | --- | --- | --- |
| **Reference group for food expenditure share** | **Q1 HH**^1^ | **Q2 HH**^1^ | **HH within 20% of the poverty line**^2^ |
|  | **Non-food expenditure (PKR/AE/day)**  **Median (SD)** | | |
| National | 63 | 91 | 87 |
|  | (22) | (26) | (27) |
| Urban Khyber Pakhtunkhwa | 66 | 92 | 90 |
|  | (19) | (24) | (25) |
| Rural Khyber Pakhtunkhwa | 65 | 92 | 88 |
|  | (21) | (26) | (27) |
| Urban Punjab | 72 | 97 | 95 |
|  | (22) | (25) | (27) |
| Rural Punjab | 66 | 91 | 88 |
|  | (21) | (27) | (28) |
| Urban Sindh | 64 | 93 | 90 |
|  | (20) | (25) | (26) |
| Rural Sindh | 55 | 84 | 80 |
|  | (20) | (25) | (26) |
| Urban Balochistan | 62 | 89 | 87 |
|  | (21) | (24) | (26) |
| Rural Balochistan | 58 | 84 | 80 |
|  | (20) | (26) | (27) |
| Source: Authors calculations using the 2018-19 Pakistan Household Integrated Economic Survey (HIES).  ^1^ Households in the first (Q1) or second (Q2) quintile in terms of total expenditure. Quintiles are computed using total expenditure at the national level and household sampling weights. Expenditure available for non-food is calculated as average share of expenditure on non-food expenses for this reference group times households’ total expenditure per capita.  ^2^ Households whose expenditures are within 20 percent of the 2018-19 poverty line = 3776 PKR per adult equivalent per month. Expenditure available for non-food is calculated as average share of expenditure on non-food expenses for this reference group times households’ total expenditure per capita. | | | |

1. Herforth AW, Holleman C, Bai Y, Masters WA. The cost and affordability of a healthy diet (CoAHD) indicators: methods and data sources: Metadata for FAOSTAT. [Internet]. 2023. Available from: https://www.fao.org/faostat/en/#data/CAHD [↑](#footnote-ref-1)
